# Hypertensive events after the initiation of contemporary cancer therapies for breast cancer control

**DOI:** 10.1002/cam4.4862

**Published:** 2022-05-27

**Authors:** Rebecca R. Carter, Aaron P. Chum, Reynaldo Sanchez, Avirup Guha, Amit K. Dey, Raquel Reinbolt, Lisa Kim, Prince Otchere, Oduro Oppong‐Nkrumah, William T. Abraham, Maryam Lustberg, Daniel Addison

**Affiliations:** ^1^ Cardio‐Oncology Program, Division of Cardiology Ohio State University Columbus Ohio USA; ^2^ The Center for the Advancement of Team Science, Analytics, and Systems Thinking (CATALYST) Ohio State University Columbus Ohio USA; ^3^ Harrington Heart and Vascular Institute Case Western Reserve University Cleveland Ohio USA; ^4^ National Heart Lung and Blood Institute Bethesda Maryland USA; ^5^ Solove Research Institute The Ohio State University Comprehensive Cancer Center – James Cancer Hospital Columbus Ohio USA; ^6^ Cancer Control Program, Department of Medicine Ohio State University Comprehensive Cancer Center Columbus Ohio USA

**Keywords:** cardio‐oncology, FDA adverse event reporting system, hypertension, national inpatient sample

## Abstract

**Background:**

Contemporary therapies improve breast cancer (BC) outcomes. Yet, many of these therapies have been increasingly linked with serious cardiotoxicity, including reports of profound hypertension. Yet, the incidence, predictors, and impacts of these events are largely unknown.

**Methods:**

Leveraging two large U.S.‐based registries, the National Inpatient Sample (NIS) and the Food and Drug Administration Adverse Event Reporting System (FAERS) databases, we assessed the incidence, factors, and outcomes of hypertensive events among BC patients from 2007 to 2015. Differences in baseline characteristics, hypertension‐related discharges, and complications were examined over time. Further, we performed a disproportionality analysis using reporting‐odds‐ratios (ROR) to determine the association between individual BC drugs and hypertensive events. Utilizing an ROR cutoff of >1.0, we quantified associations by drug‐class, and individual drugs with the likelihood of excess hypertension.

**Results:**

Overall, there were 5,464,401 BC‐admissions, of which 46,989 (0.8%) presented with hypertension. Hypertensive BC patients were older, and saw initially increased in‐hospital mortality, which equilibrated over time. The mean incidence of hypertension‐related admissions was 732 per 100,000 among BC patients, versus 96 per 100,000 among non‐cancer patients (RR 7.71, *p* < 0.001). Moreover, in FAERS, those with hypertension versus other BC‐treatment side‐effects were more frequently hospitalized (40.1% vs. 36.7%, *p* < 0.001), and were most commonly associated with chemotherapy (45.9%). Outside of Eribulin (ROR 3.36; 95% CI 1.37–8.22), no specific drug was associated with a higher reporting of hypertension; however, collectively BC drugs were associated with a higher odds of hypertension (ROR 1.66; 95% CI 1.09–2.53).

**Conclusions:**

BC therapies are associated with a substantial increase in limiting hypertension.

## Graphical abstract

Leveraging two large U.S.‐based registries, the National Inpatient Sample (NIS) and the Food and Drug Administration Adverse Event Reporting System (FAERS) databases, we assessed the incidence, factors, and outcomes of hypertensive events among breast cancer patients from 2007‐2015. We found that breast cancer therapies associate with a substantial increase in limiting hypertension.

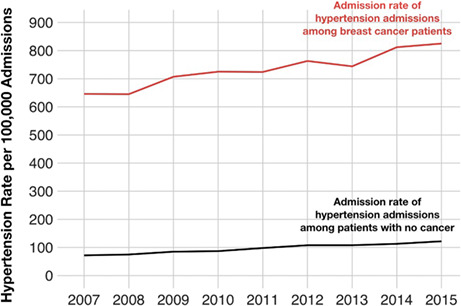

## 1 INTRODUCTION

In the United States (U.S.), nearly 1 in 8 women will go on to develop breast cancer.^1^ Fortunately, in the era of contemporary anticancer therapies, expected survival now approaches 80%.^1^ However, with increasing survival, breast cancer patients' non‐malignant conditions, such as cardiovascular disease (CVD), have been more frequently recognized as the primary drivers of long‐term morbidity and mortality.^2^, ^3^ Furthermore, emerging cancer therapies have been shown to have increasing adverse cardiotoxicities which adds to the cardiovascular burden in this population.^4^, ^5^ Although initial clinical studies were limited in their ability to describe these events, their timing, and general effects have been increasingly well defined.

However, increasingly, many of these cancer therapies have been recognized to associate with more insidious subclinical forms of incident CVD, including hypertension.^4^, ^5^, ^6^, ^7^, ^8^, ^9^, ^10^ Available data have generally suggested a prevalence of hypertension of up to 13% within 2 years of anticancer therapy initiation among selected breast cancer populations.^6^, ^7^, ^8^ Yet, as many prior therapeutic clinical trials either excluded hypertensive patients or may have overlooked the occurrence of hypertension during trial completion, the true incidence of these events is largely unclear.^9^, ^10^, ^11^, ^12^ Despite consistent evidence characterizing the link between contemporary anticancer therapies and hypertension among less common forms of cancer, the exact nature of the association between breast cancer therapies and hypertension or its ramifications in real‐world clinical care remain unknown.^13^

In this study, we evaluated the baseline characteristics and predictors of hypertensive events among breast cancer patients using two national registries that captured and reflected contemporary practice. We also sought to explore whether breast cancer was associated with a higher prevalence of hypertension‐related hospitalizations, and the potential factors underlying the development of these potentially limiting events.

## 2 METHODS

### 2.1 Data sources—NIS and FAERS

The National Inpatient Sample (NIS) is a large administrative database of all‐payer health care utilization produced by the Agency for Healthcare Research and Quality (AHRQ) and is the largest publicly available all‐payer inpatient database in the U.S. Developed under the AHRQ Healthcare Cost and Utilization Project (HCUP), NIS includes administrative and demographic data from a 20% sample of discharges among all U.S. HCUP hospitals—excluding rehabilitation and long‐term acute care hospitals—equating approximately 8 million hospital stays from over 1000 hospitals. This study queried NIS hospital discharge datasets from January 2007 through December 2015.

The U.S. Food and Drug Administration Adverse Event Reporting System (FAERS) is a publicly available, spontaneous reporting system that contains data on adverse drug events and medication errors submitted to the Food and Drug Administration (FDA). The FAERS contains data generated by reports from healthcare professionals and patients, as well as drug manufacturers for serious adverse reactions. The information is provided as free text and adverse drug events are coded with the Medical Dictionary for Regulatory Activities (MedDRA). All reports available in the FAERS database from Quarter 1, 2007 to Quarter 4, 2015 were accessed and downloaded from the FDA website (http://www.fda.gov/).

### 2.2 Study population—NIS

We identified all hospitalized adults (≥18 years) among the NIS cohort based on the International Classification of Diseases, Ninth Revision (ICD‐9), who had a primary diagnosis code (*DX1*) for hypertension (174.x − 175.x) and at least 1 additional diagnosis code for breast cancer (401.x–405.x); near 99% were female. Hypertension typically reflected values deemed high enough by the treating provider to impact care and/or require hospitalization. Patients were considered non‐cancer (female) patients if they did not have a cancer Diagnosis Clinical Classification System (DXCCS) code or a cancer comorbidity code. We selected female controls, as the vast majority breast cancer is seen women.^1^ NIS variables included in the study were demographic characteristics (i.e., age and race), length of stay, and co‐morbidities used to calculate an Elixhauser's Comorbidity score (i.e., a set of 31 co‐morbidities computed from ICD‐9 codes). Individual patients cannot be tracked, however, NIS provides a unique identifier to link records across time between the Inpatient Core file, the Hospital Weights, and Disease Severity Measures.

### 2.3 Study population—FAERS

We identified all adults (≥18 years) among FAERS cohort extraction of drug‐hypertension event pairs among patients presenting with breast cancer indication. We implemented natural language processing (NLP) techniques to pre‐process drug information captured in the unstructured text of the database. NLP strategies included text mining with entity recognition, relation detection, word sense disambiguation, and temporal inference.^14^, ^15^, ^16^ With the located and classified named drug entities, we then mapped trade names to generic names. Breast cancer drugs were partitioned into seven breast cancer classes according to the targeted receptors of breast cancer subtypes: Chemotherapy, human epidermal growth factor receptor 2 (HER2) Targeted, Aromatase Inhibitors, Hormones, Biologic (other than HER2 targeted agents), Immunotherapy, and Other Targeted. Available demographics were age, gender, and weight (converted to kilograms where appropriate). Individual patients and their treatment regimens cannot be tracked over time with this data source, as its records consist of only spontaneous reports of drug‐related adverse events.

### 2.4 Outcome measures

Within NIS, our primary outcome was the report of clinical hypertension events (hospitalizations), by breast cancer status. NIS provided data on specific outcomes including length of stay and discharge disposition. The inpatient hospital charges were converted to estimated costs using the HCUP‐provided cost to charge ratios and wage index for a given year. The wage index corrects for geographic variations in costs among hospitals. Estimates were inflation adjusted to 2015 U.S. dollars using annual hospital consumer‐price indices data from the U.S. Bureau of Labor Statistics.^17^ From FAERS, our outcomes focused on relative odds of reported hypertensive events after BC therapy initiation. We also considered the odds of adverse events, including hospitalization, life‐threatening disability, and/or death, by hypertension status.

### 2.5 Statistical analysis

Differences in baseline characteristics, hypertension‐related discharges, and complications were examined in each cohort over time with parametric and non‐parametric approaches, including both paired and unpaired *t*‐tests, and χ^2^ tests. Categorical variables are presented as N (%) and continuous variables are presented as mean ± standard deviation (SD). We determined the association between each of the aforementioned categorical demographic and patient factors with each of the aforementioned complications and hypertension‐related discharge using χ^2^ tests. Odds ratios (ORs) with 95% confidence intervals (CI) and their associated *p* values are presented for each of these analyses.

For NIS, we used survey analysis techniques to account for data clustering and stratification. This leveraged established bias‐corrected weighted estimate equations for complex data, to model hypertension outcomes a function of covariates.^18^ Further, the annual HCUP‐provided discharge weights were incorporated to allow for nationally representative population weighting of discharges. To determine which demographic and patient factors were most predictive of hypertension, we performed a multivariable logistic regression model adjusting for patient age, gender, race, and Elixhauser comorbidity score. The Elixhauser comorbidity score considers 31 potential comorbidities based on diagnosis codes; and accounts for risk factors commonly linked with cardiovascular and other adverse events, length of stay, and in‐hospital mortality.^19^ Additionally, we performed a sensitivity analysis by repeating our analyses with a subgroup without cardiovascular disease risk factors and history. Specifically, we excluded patients with a diagnosis code for renal failure (*N* = 196 H‐HTN, *N* = 10,889 NH‐HTN), diabetes (*N* = 112 H‐HTN, *N* = 8325 NH‐HTN), stroke (*N* = 55 H‐HTN, *N* = 4394 NH‐HTN), and myocardial infarction (*N* = 18 H‐HTN, *N* = 2723). In instances of age and comorbidity, our conclusions remained the same (Table S2).

For FAERS, spontaneous adverse events (AEs) of hypertension associated with breast cancer drugs were analyzed using the reporting odds ratio (ROR), a measure to highlight disproportionally reported signals of spontaneous adverse drug reaction reports in FAERS from Quarter 1, 2007 to Quarter 4, 2015. To calculate ROR, a 2 × 2 contingency table was drawn for each association between hypertension and a drug of interest (Table S1).^20^ The total number of adverse event reports for a given drug was obtained by searching the entire database using drugs associated with hypertension reports. For each ROR, two‐sided 95% CIs were calculated by the Mantel–Haenszel test. A ROR value of 1 suggests that there is neither a disproportional association nor dissociation of drug. A signal for alarm was ROR >1, indicating that the drug of interest had greater odds of being reported for hypertension than other adverse events.^20^, ^21^

We plotted summary statistics for clinical outcomes and associated drug classes over the selected 9‐year period. A *p* value of ≤0.01 was considered significant. All statistical analyses were performed with R software, version 3.5.1 (R Foundation for Statistical Computing); packages utilized included cluster, odds ratio, sensitivity, survey, survival, and tidyverse. This study was deemed institutional review board exempt: Existing data from a large database such as NIS was collected in such a manner that subjects could not be identified directly or through identifiers linked to the subjects and FAERS data are publicly available.

## 3 RESULTS

### 3.1 Incidence and patterns of profound hypertension in breast cancer patients—NIS

Within the NIS cohort overall there were 5,464,401 breast cancer admissions, of which 46,989 (0.80%) presented with hypertension. The overall weighted average prevalence of hypertension‐related admissions was 732 per 100,000 admissions, compared to 96 among non‐cancer patients (RR 7.71; 95% CI 7.64–7.79; *p* < 0.001). Breast cancer patients were older (mean: 70 years, SD: 14.0), more commonly men, and reported a median Elixhauser comorbidity score of 18 (SD = 15.1) (Table 1). In addition, older breast cancer patients and those with comorbidities saw higher rates of hypertension‐related admissions (OR: 1.40; 95% CI 1.19–1.65; *p* < 0.001 and OR: 1.02; 95% CI 1.016–1.03; *p* < 0.001, respectively). In comparison to white women, black and Hispanic breast cancer patients saw higher rates of hypertension‐related admissions (OR: 2.44; 95% CI 2.04–2.91; *p* < 0.001 and OR: 1.52; 95% CI 1.12–2.03; *p* < 0.001, respectively) (Table 2).

**TABLE 1:** Characteristics of breast cancer patients hospitalized with hypertension*

Patient characteristics	Hospitalized with hypertension
**NIS data (2007–2015)**	
Age, years (Mean ± SE)	70 (14.0)
Race/Ethnicity	
White	48.0
Black	36.5
Hispanic	6.6
Outcome	
Death	0.3
Stroke	0.9
Discharge disposition	
Home/Routine	67.1
Home Health Care	16.4
Transferred	16.0
Average Length of Stay in Days (Mean ± SE)	4.6 (5.0)
Total Elixhauser Comorbidity Score (Mean ± SE)	22.4 (15.8)
**FAERS Data (2007–2015)**	
Age in Years (Mean ± SE)	56.5 (12.7)
Outcome	
Death	16.2
Hospitalization	40.1
Disability	2.4
Breast Cancer Drug Class	
Chemotherapy	43.6
HER2 targeted	22.1
Aromatase inhibitors	13.8
GNRH Agonist	1.1
Biological	6.6
Hormone	6.1
Immunotherapy	3.9
Other targeted	2.8

Abbreviations: BC, breast cancer; GNRH, gonadotropin‐releasing hormone; HER2, human epidermal growth factor receptor 2; SE, standard deviation.

* Patient‐level cancer stage was not available.

**TABLE 2:** Multivariable model of patients with underlying breast cancer admitted for hypertension vs. other causes*

		Est.	SE	ROR*	CI	*p*‐value
**(Intercept)**		−6.06	0.135	0.0023	0.002–0.003	<0.0001
**Age** (REF: <65 years)		0.33	0.08	1.40	1.19–1.65	<0.0001
**Elixhauser comorbidity score**		0.02	0.002	1.02	1.016–1.03	<0.0001
**Length of stay group**	1–3 Days	0.19	0.10	1.22	0.99–1.50	=0.05
(REF: 7 days or more)	4–6 Days	0.03	0.12	1.03	0.82–1.30	‐
**Race**	Asian	−0.29	0.34	0.75	0.36–1.37	
(REF: white)	Black	0.89	0.09	2.44	2.04–2.91	<0.0001
	Hispanic	0.42	0.15	1.52	1.12–2.03	<0.001

Abbreviations: CI, confidence interval; Est, estimate.

* Adjusted for age, gender, race, and Elixhauser comorbidity score.

**FIGURE 1:**
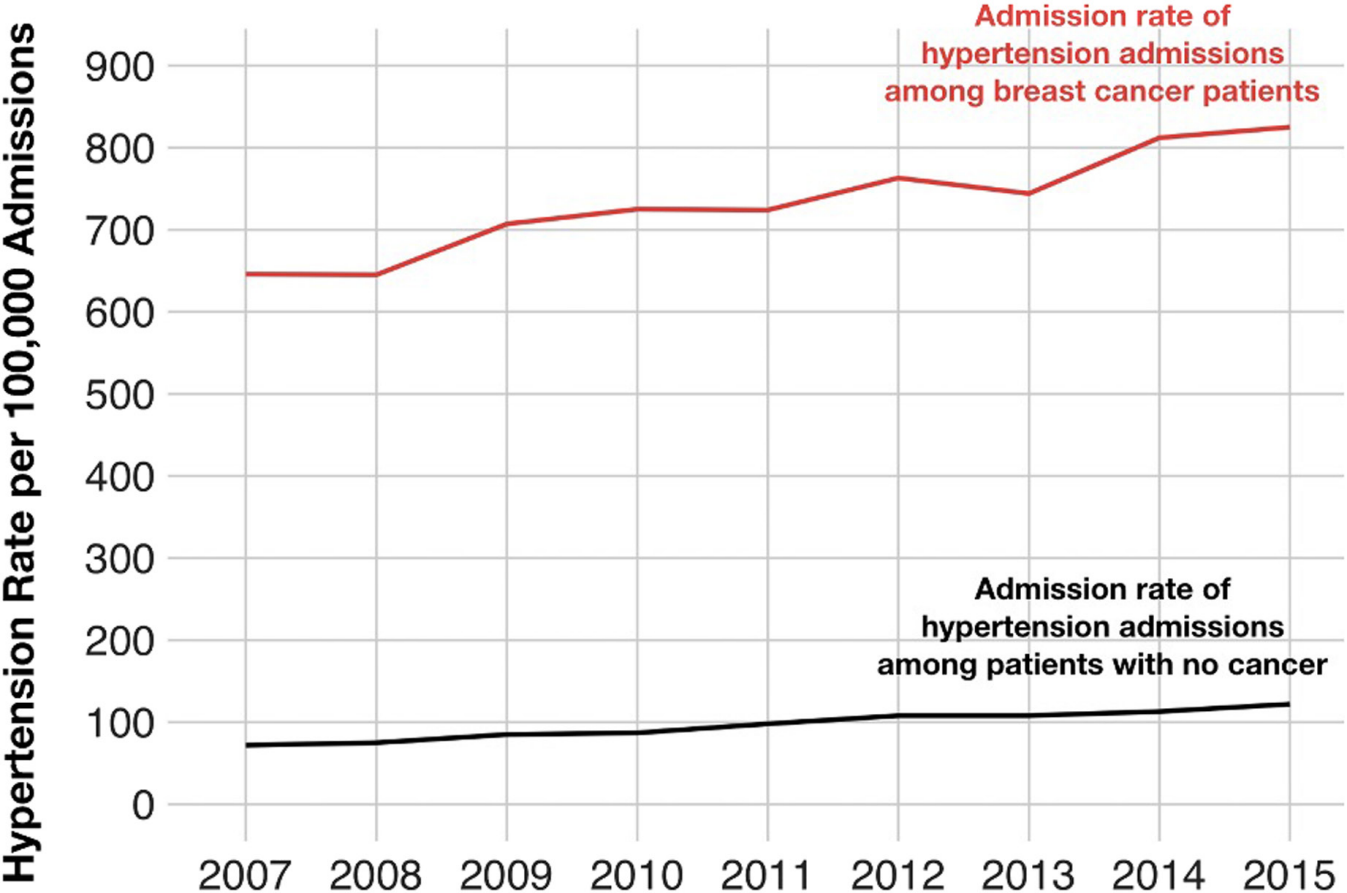
Admission rate for hypertension in female breast cancer patients compared to the admission rate for hypertension in patients with no cancer in NIS (2007–2015).

Over time, hypertension admission rates among cancer patients increased, along with a mild concurrent increase among non‐cancer patients. From 2007 to 2015 there was a relative increase of 26.4% hypertension‐related admissions per 100,000 breast cancer patients compared to 9.2% of hypertension admissions per 100,000 non‐cancer patients (Figure 1). This trend was most evident in the more recent available years studied. Moreover, this increasing trend in hypertension‐related admissions among cancer patients was also noted in subgroup analyses of younger‐aged patients (age < 50 years) when compared to their counterparts.

### 3.2 Reporting patterns of hypertension with breast cancer therapies over time—FAERS

Next, we sought to explore the association of type of anti‐cancer therapy with hypertension‐related events in breast cancer. Those with hypertension and breast cancer‐specific therapies were more frequently hospitalized (40.1% vs. 36.7%, *p* < 0.001) compared to those without hypertension noted. The total number of adverse events in FAERS from all breast cancer drugs was 44,798 (Table 3). Of those adverse events, 181 were hypertension‐related in nature.

**TABLE 3:** Most frequent breast cancer therapies associated with hypertension, by drug class across time (in 2007, 2015, and 2007 through 2015)

	2007	2015	2007–2015
**Chemotherapy**	Capecitabine	Docetaxel	Capecitabine
**HER2 targeted**	Trastuzumab	Trastuzumab	Trastuzumab
**Aromatase inhibitor**	Anastrozole	Letrozole	Anastrozole
**Hormone**	–	–	Tamoxifen
**Other targeted**	–	Palbociclib	Palbociclib
**Biologic**	–	–	Bevacizumab
**Immunotherapy**	–	Everolimus	–

Abbreviations: HER2, human epidermal growth factor receptor 2.

**FIGURE 2:**
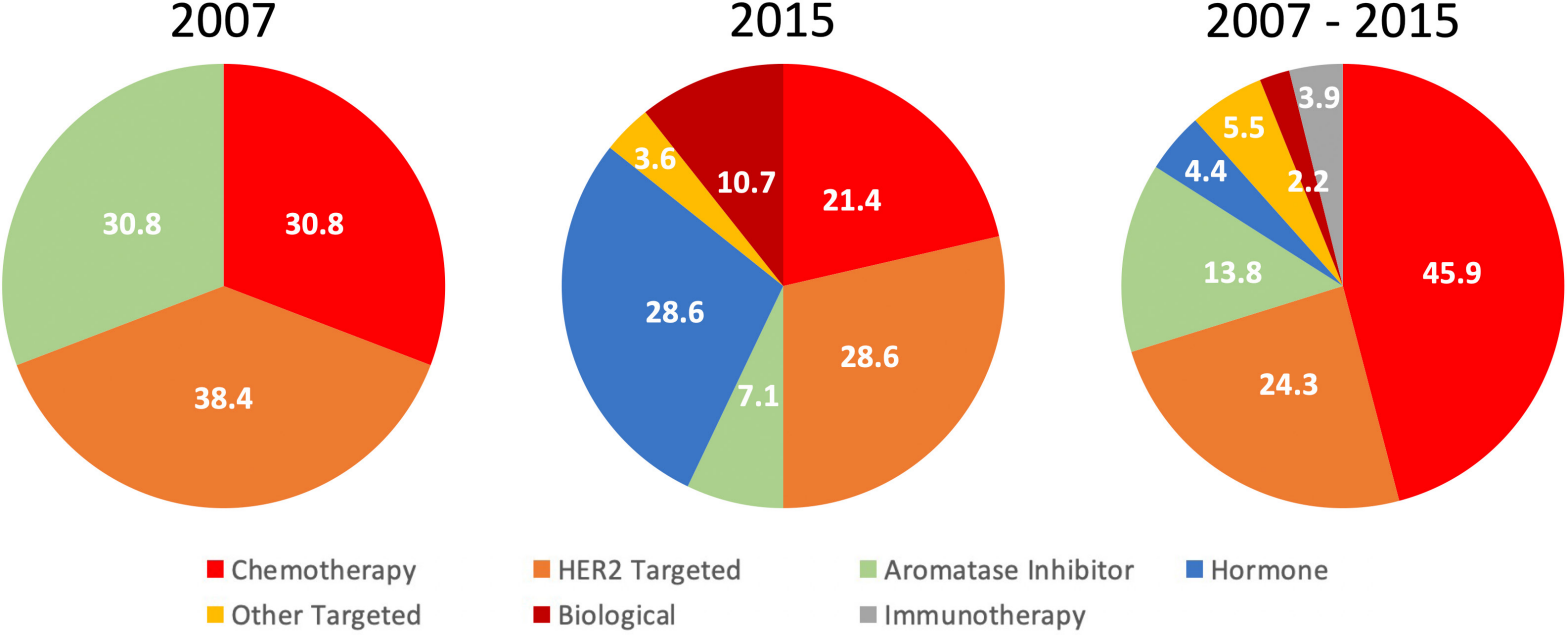
Representative percentage of female breast cancer hypertension reports in FAERS attributable to each therapy class in 2007, and 2015, as well as across the 2007–2015 period, respectively.

Nearly half of breast cancer therapies associated with hypertension were considered as chemotherapeutics (45.9%, *N* = 83; Figure 2). This was followed by HER2 Targeted drugs (24.3%, *N* = 44). Overall, the HER2 Targeted drug trastuzumab had the most frequent hypertension‐related adverse events from 2007 through 2015, followed by the monoclonal antibody bevacizumab (Table S3). Although trastuzumab had an elevated ROR of 1.28, regarding overall adverse events it was more likely to be associated with hypertension‐related adverse side effects compared to all other adverse side effects (27 reports; 95% CI 0.81–1.93; Table S4). Additionally, the combined ROR for the top 20 most commonly reported breast cancer drugs was 1.66 (156 reports; 95% CI 1.09–2.53), indicating greater odds of being reported for a hypertension‐related adverse event compared to all other adverse events. This relationship may be driven in majority by eribulin, which had one of the highest hypertension‐related adverse event ROR irrespective of drug class (5 reports; ROR 3.36, 95% CI 1.37–8.82). Moreover, over time hypertension‐related events associated with overall breast cancer therapies increased in number from 2007 through 2015 (*p* < 0.001), and this was largely driven by the chemotherapy arm (Table 3).

### 3.3 Hypertension and in‐hospital outcomes among breast cancer patients

Within NIS, overall unadjusted in‐hospital mortality rates followed a decreasing trend among all groups over an extended period of time from 2003 to 2015 (*p*‐trend for breast cancer <0.001 as well as non‐cancer group *p* < 0.001, respectively); but was unchanged over the 2007 to 2015 period. However, despite breast cancer patients seeing higher initial mortality, the overall risk of in‐hospital mortality remained similar among those with hypertension and underlying breast cancer over time versus those without cancer (3.1% for breast cancer in 2003 and 2.0% for non‐cancer in 2003, respectively; versus 1.3% for breast cancer in 2015 and 1.4% for non‐cancer 2015, respectively; *p* = 0.73; Figure S1). Further, there was no difference in the lengths of stay for hypertension‐related admissions among breast cancer patients (for the year 2015: breast cancer = 2.9 (SE 0.13) versus non‐cancer = 2.6 (SE 0.03) and *p* = 0.69) compared to hypertension‐related admissions among non‐cancer patients at the index visit. However, nearly 82.6% of hypertension‐related hospitalizations in breast cancer patients compared with 56.9% in the non‐cancer patients, were billed to Medicare/Medicaid (*p* < 0.001). These trends were consistent over the period considered.

## 4 DISCUSSION

In this evaluation of two contemporary national cohorts, we found breast cancer patients to be over seven times more likely to be hospitalized for hypertension than non‐cancer patients. This was more pronounced in older patients and those with comorbidities. Moreover, among those with breast cancer and presenting with significant hypertension, treatment with the 20 most commonly utilized breast cancer agents was likely to see an increase in reported hypertensive‐related events. Collectively, our analyses were the first to use two contemporary national registry data to evaluate the real‐world association between hypertension and breast cancer. Given the ubiquitous use of these agents in clinical practice to treat breast cancer, appreciation of the increasing rates of hypertension may bear weight on the understanding of cardiovascular disease among this rapidly growing population.

The proliferation of anticancer therapies over the last two decades has led to prolonged survival in BC patients, necessitating a heightened focus on the downstream effects of successful treatment, including CVD.^2^, ^3^, ^22^ This has been paralleled by increases in more insidious forms of CVD, inclusive of hypertension seen with the use of emerging therapies. Prior analysis from populations treated primarily with anthracycline‐based regimens in the 1990s through the early 2000s have suggested a signal of increased hypertension. However, this has remained relatively understudied. With nascent waves of anticancer therapies garnering FDA approval, the relevance of hypertension has increasingly come into question.^23^ Often in practice, hypertension, or its consequences, are not well appreciated by oncologic and CVD providers. Drawing from the diverse and large sample size captured in the NIS and FAERS databases, these current data offer clarity and insight into the scope of even profound hypertension among growing BC populations.

The development or worsening of hypertension following initiation of anticancer therapy has been increasingly recognized as an underappreciated link to subsequent major CVD in the presence of cancer.^24^, ^25^, ^26^, ^27^ This is relevant as recent studies by pivotal Systolic Blood Pressure Intervention Trial (SPRINT) investigators have advocated that strict blood pressure control has incremental benefit in relation to incident cardiovascular events compared to standard blood pressure control.^28^, ^29^ Moreover, prior studies suggest that the presence of CVD risk factors such as hypertension among breast cancer patients during treatment may critically predict the development of major CVD events associated with not only cancer but also with the use of anticancer agents.^30^ Our results imply that focusing on blood pressure management after initiation of anticancer treatment may be relevant to prevent insidious CVD and the need for unexpected and/or complicated hospital admissions. This is in line with recent recommendations regarding aggressive blood pressure monitoring and treatment for hypertension with antihypertensive medication in patients receiving breast cancer therapy, which were recommended by an expert panel of the National Cancer Institute.^31^ And although there are no prospective cancer‐focused studies evaluating the effects of blood pressure control, available non‐cancer data suggest a potential basis for efficacy. Furthermore, in the general population, black women have more CVD risk factors such as hypertension compared to white women of the same age, a phenomenon observed within this BC‐focused population.^32^ Black women may be more vulnerable to a higher risk of morbidity and mortality from hypertension‐related events following treatment for breast cancer.^33^ Globally, BC patients are exposed to a series of sequential or concurrent adverse events (e.g., increased inflammation) that together make them more vulnerable to the development of hypertension, and other CVD events.^34^, ^35^ Thus, given these potential ramifications of hypertension, enhanced study, vigilance, and blood pressure control may prove pivotal. However, additional prospective BC‐focused studies are needed.

### 4.1 Limitations

Several limitations should be acknowledged. NIS is an administrative database, and identification of cases was based on a combination of ICD‐9 codes. The study may be subject to changes in coding practices and miscoding; however, the sample size (i.e. over 5,000,000 admissions) is large enough to compensate for those errors. The dataset also does not allow for precise staging of cancer, beyond the presence of advanced or metastatic disease, nor is there provision for ongoing anticancer therapy, timing, or type of anticancer therapy (e.g., targeted vs. traditional), or code status. Use of more than one drug at a time could not be delineated. Spontaneous reporting system databases like FAERS are also distinguished by some important limitations, most notably no definitive proof of the causal relationship between exposure to the drug and the reported adverse event.^36^ Further, FAERS relies heavily upon voluntary reporting, therefore the frequency of actual hypertension events is very likely to be underestimated, or influenced by the “Weber Effect,” where reporting of adverse event is biased to the end of the 2nd year post‐regulatory approval, and “stimulated reporting”, indicating increased reports due to alerts issued by the FDA.^37^, ^38^, ^39^ Data on preceding hypertension was not available. The time to event for all cases with adverse events was not available, nor was the grade of toxicity and adverse event reporting for a drug may be influenced by extent of use, publicity, and bias. In addition, the higher prevalence of hypertension in the general inpatient population (~50%) may mean that the effect estimate of the inpatient breast cancer population may be higher than for breast cancer patients in the outpatient setting.^32^ BC stage or metastasis data were not consistently available. The relationship between tamoxifen and CVD is mixed.^22^, ^34^ Finally, neither the incidence of adverse events nor the absolute measures of risk can be computed from the analyses of FAERS due to the lack of denominator (i.e. the number of patients prescribed the drug).^40^, ^41^

## 5 CONCLUSIONS

In the era of contemporary cancer therapies, limiting hypertension is an increasingly common limitation of effective anticancer therapy. Among breast cancer patients, common anticancer strategies associated with a significantly increased risk of profound hypertension, even leading to a greater than sevenfold increased need for hospitalizations. The occurrence of these events is associated with increased morbidity. These effects were most evident in older and minority populations. Given the expected continued rise in the number of breast cancer therapies, enhanced evaluation, and vigilance for the detection of hypertension, and effective blood pressure controlling strategies, are needed.

## AUTHOR CONTRIBUTIONS

*Concept and design:* RC, RS, AG, DA.

*Acquisition, analysis, or interpretation of data:* RC, AC, RS, AG, AD, LK, DA.

*Drafting of the manuscript:* RC, AC, RS, AG, AD, DA.

*Critical revision of the manuscript for important intellectual content:* All authors.

*Statistical analysis:* RC, AC, AG, PO, OON, DA.

*Supervision:* DA.

## Funding information

Dr. Addison was supported by NIH grant number K12‐CA133250, K23‐HL155890, and by a Robert Wood Johnson Foundation (Harold Amos)—American Heart Association Faculty Development Program grant. Dr. Carter was supported by the Mary H. and J. Churchill Hodges Clinical Prevention Program Fund at the Ohio State University Wexner Medical Center.

## DISCLOSURES

All other authors declare no conflicts of interests in relation to the work presented in this manuscript.

## DATA SHARING POLICY

The data that support the findings of this study are available from the corresponding author upon reasonable request.

## Supporting information


Figure S1

Table S1‐S4

## Data Availability

Data Sharing Policy: The data that support the findings of this study are available from the corresponding author upon reasonable request.
