# Supplementary figures and images for: Hypertensive events after the initiation of contemporary cancer therapies for breast cancer control
Source: Cancer Med. 2022 May 27;12(1):297–305. doi: 10.1002/cam4.4862 (PMC9844596; doi:10.1002/cam4.4862)

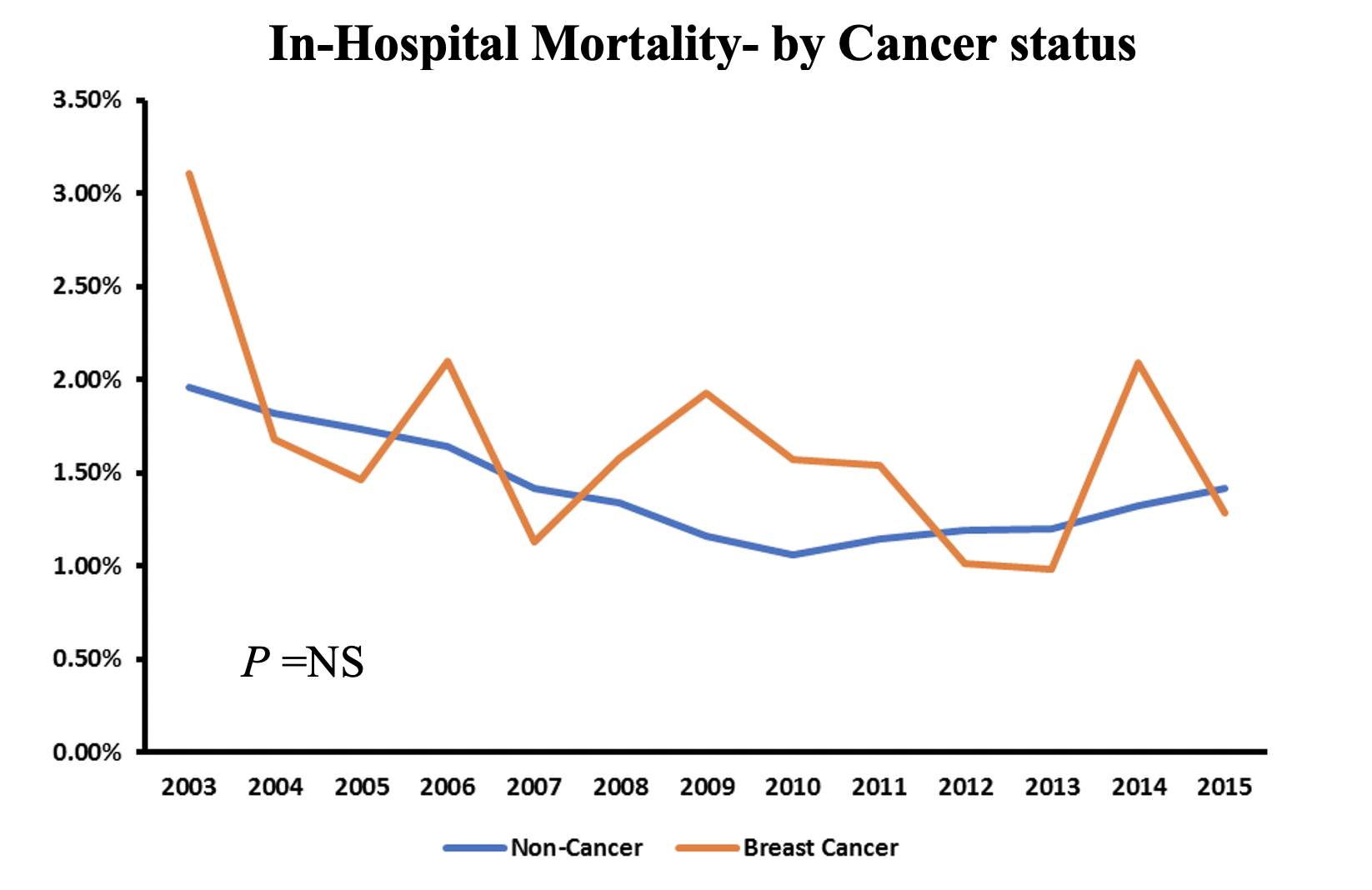

Supplement: Supplementary file 1 — Figure S1 [file CAM4-12-297-s001.png]
