# Supplementary material for: Hypertensive events after the initiation of contemporary cancer therapies for breast cancer control
Source: Cancer Med. 2022 May 27;12(1):297–305. doi: 10.1002/cam4.4862 (PMC9844596; doi:10.1002/cam4.4862)
Supplement: Supplementary file 2 — Table S1‐S4 [file CAM4-12-297-s002.docx]

**Supplemental Table 1.** A 2x2 contingency table for calculating disproportionality. The cell *a* represents the number of reports on which the same combination of Breast Cancer (BC) drug and hypertension were mentioned, cell *b*, the number of reports concerning the BC drug but with other possible reported events, cell *c* the number of reports concerning the adverse events associated with other drugs, and cell *d* the number of reports concerning other drugs associated with other adverse events. The reporting odds ratio (ROR) is defined as the product of the exposure odds among the cases in respect to the exposure odds among the non-cases and is calculated by the equation below:

|  | **Reports with hypertension** | **Reports without hypertension** |
| --- | --- | --- |
| Reports with the drug of interest | *a* | *b* |
| All other reports | *c* | *d* |

ROR = $\frac{\text{a}\text{ }\times\text{d}}{\text{b}\text{ × }\text{c}}$

**Supplemental Table 2.** Sensitivity analysis for the risk of incident event among breast cancer patients, excluding those with pre-existing CVD.

|  | | **Est.** | **Std. Error** | **Odds** | **CI** | **P-Value*** |
| --- | --- | --- | --- | --- | --- | --- |
| **(Intercept)** | | -6.95 | 0.52 | 0.001 | 0.0003-0.0025 | <0.0001 |
| **Age Group** (REF:  <65 Years) | | 0.74 | 0.37 | 2.10 | 1.05-4.50 | <0.01 |
| **Elixhauser Comorbidity Score** | | 0.02 | 0.008 | 1.03 | 1.01 – 1.04 | <0.001 |
| **Length of Stay Group** | 1-3 Days | -0.10 | 0.40 | 0.91 | 0.40-2.01 | **-** |
| (REF: 7 Days or more) | 4-6 Days | 0.24 | 0.37 | 1.27 | 0.61-2.68 | - |
| **Race** | Asian | -13.99 | 0.34 | 0.75 | 0.36-1.37 | - |
| (REF: White) | Black | 0.32 | 0.09 | 1.38 | 0.57-3.00 | - |
|  | Hispanic | 0.69 | 0.15 | 2.01 | 0.59-5.22 | - |

Abbreviations: CI, confidence interval; CVD, cardiovascular disease; Est, estimate. * “-” a non-significant P-value.

**Supplemental Table 3:** Reporting odds ratios for the top 20 most frequently reported breast cancer drugs from 2007 through 2015 in FAERs.

| **Breast Cancer  Drug Class** | **Breast Cancer Drug** | **No. of all Hypertension reports** | **No. of all adverse event reports** | **ROR (95% CI)** |
| --- | --- | --- | --- | --- |
| HER2 Targeted | Trastuzumab | 27 | 5414 | 1.28 (0.81-1.93) |
| Chemotherapy | Bevacizumab | 12 | 2831 | 1.05 (0.58 -1.89) |
| Chemotherapy | Capecitabine | 10 | 2520 | 0.98 (0.52 - 1.86) |
| HER2 Targeted | Lapatinib | 10 | 2371 | 1.05 (0.55 - 1.98) |
| Aromatase Inhibitor | Anastrozole | 9 | 2613 | 0.84 (0.43 - 1.65) |
| Aromatase Inhibitor | Letrozole | 9 | 1607 | 1.41 (0.72 - 2.76) |
| Chemotherapy | Docetaxel | 9 | 2206 | 1.01 (0.52 - 1.98) |
| Chemotherapy | Epirubicin | 8 | 1739 | 1.15 (0.56 - 2.33) |
| Aromatase Inhibitor | Exemestane | 7 | 1563 | 1.11 (0.52 - 2.37) |
| Chemotherapy | Paclitaxel | 7 | 2703 | 0.63 (0.29 - 1.33) |
| Chemotherapy | Doxorubicin | 6 | 1183 | 1.27 (0.56 - 2.86) |
| Hormone | Tamoxifen | 6 | 944 | 1.60 (0.71 - 3.61) |
| Chemotherapy | Eribulin | 5 | 379 | 3.36 (1.37 - 8.22) |
| Other Targeted | Palbociclib | 5 | 600 | 2.10 (0.86 - 5.13) |
| Chemotherapy | Carboplatin | 4 | 890 | 1.12 (0.30 - 2.92) |
| Chemotherapy | Cyclophosphamide | 4 | 2105 | 0.46 (0.17 - 1.23) |
| Chemotherapy | Taxol | 4 | 586 | 1.71 (0.63 - 4.62) |
| Immunotherapy | Everolimus | 4 | 1263 | 0.79 (0.29 - 2.10) |
| Immunotherapy | Endoxan | 4 | 1055 | 0.94 (0.35 - 2.53) |
| HER2 Targeted | Pertuzumab | 6 | 833 | 1.82 (0.80 - 4.11) |
| Top 20 BC drugs combined | | 156 | 35405 | 1.66 (1.09 - 2.53) |

Abbreviations: BC, breast cancer; HER2, human epidermal growth factor receptor 2; ROR, reporting odds ratio.

**Supplemental Table 4:** Reporting odds ratios by breast cancer class from 2007 through 2015 in FAERS.

| **Overall Breast Cancer  Drug Classes** | **No. of all Hypertension Reports** | **No. of all adverse event reports** | **ROR (95% CI)** |
| --- | --- | --- | --- |
| Chemotherapy | 83 | 23161 | 0.79 (0.59 - 1.06) |
| HER2 Targeted | 44 | 9522 | 1.19 (0.85 -1.67) |
| Aromatase Inhibitor | 25 | 6158 | 1.01 (0.66 - 1.54) |
| Hormone | 8 | 1474 | 1.36 (0.67 - 2.77) |
| Other Targeted | 10 | 2436 | 1.02 (0.54 - 1.93) |
| Biological | 4 | 738 | 1.35 (0.50 - 3.65) |
| Immunotherapy | 7 | 1309 | 1.34 (0.63 - 2.85) |
| All BC drug classes combined | 181 | 44798 | 0.66 (0.39 – 1.10) |

Abbreviations: BC, breast cancer; CI, confidence interval; FAER, Food and Drug Administration adverse event reporting system; HER2, human epidermal growth factor receptor 2; ROR, reporting odds ratio.
